# Face Inversion Reduces the Persistence of Global Form and Its Neural Correlates

**DOI:** 10.1371/journal.pone.0018705

**Published:** 2011-04-15

**Authors:** Lars Strother, Pavagada S. Mathuranath, Adrian Aldcroft, Cheryl Lavell, Melvyn A. Goodale, Tutis Vilis

**Affiliations:** 1 Centre for Brain and Mind, University of Western Ontario, London, Ontario, Canada; 2 Department of Physiology and Pharmacology, University of Western Ontario, London, Ontario, Canada; 3 Department of Psychology, University of Western Ontario, London, Ontario, Canada; 4 Department of Neurology, Sree Chitra Tirunal Institute for Medical Sciences and Technology, Trivandrum, India; Monash University, Australia

## Abstract

Face inversion produces a detrimental effect on face recognition. The extent to which the inversion of faces and other kinds of objects influences the perceptual binding of visual information into global forms is not known. We used a behavioral method and functional MRI (fMRI) to measure the effect of face inversion on visual persistence, a type of perceptual memory that reflects sustained awareness of global form. We found that upright faces persisted longer than inverted versions of the same images; we observed a similar effect of inversion on the persistence of animal stimuli. This effect of inversion on persistence was evident in sustained fMRI activity throughout the ventral visual hierarchy, including the lateral occipital area (LO), two face-selective visual areas—the fusiform face area (FFA) and the occipital face area (OFA)—and several early visual areas. V1 showed the same initial fMRI activation to upright and inverted forms but this activation lasted longer for upright stimuli. The inversion effect on persistence-related fMRI activity in V1 and other retinotopic visual areas demonstrates that higher-tier visual areas influence early visual processing via feedback. This feedback effect on figure-ground processing is sensitive to the orientation of the figure.

## Introduction

Face inversion produces a detrimental effect on recognition [1], and this effect is thought to reflect a failure of configural (or ‘holistic’) processing [2], [3], [4], [5]—the binding of facial features into a unified perceptual representation [6]. Neurophysiological studies have shown that face inversion influences visual processing during the first 170 ms of visual processing [7], [8], [9], [10], and several functional magnetic resonance imaging (fMRI) studies suggest that the fusiform face area (FFA) [11] is the neural basis of the face inversion effect on recognition [12], [13], [14], [15], [16]. These findings are consistent with increasing consensus that the face inversion effect originates during perceptual encoding rather than long-term memory [17], [18], [19], [20]. However, the extent to which face inversion and other object inversion effects influence the visual perception of form is not known.

**Figure 1:**
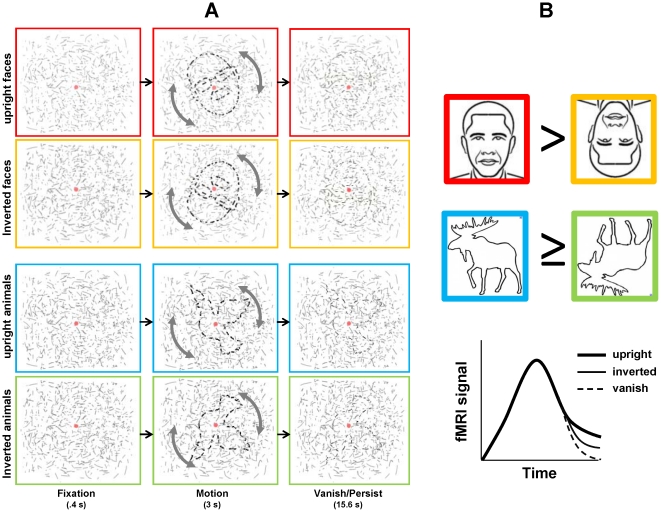
Experimental design and predictions. (A) Conditions and trial sequence. Figures became visible during Motion phase. In the Persist condition, the line segments comprising these figures remained superimposed on the background elements and were perceived to fade gradually into a background of similar lines; in the Vanish condition these line segments were removed and the figure disappeared abruptly (line segments corresponding to the figure have been darkened here for purposes of illustration; see text for details). (B) We predicted that persistence would be longer for upright faces as compared to inverted faces (red and yellow icons, respectively) we were uncertain whether or not inversion would influence the persistence of animals similarly (blue and green icons). Icons are not representative of actual figure stimuli (see Figure S1). We expected that our fMRI results (lower right) would reflect the behavioral inversion effect such that upright faces (and possible animals) in the Persist condition would show a more gradual decay of fMRI signal than inverted counterparts.

In addition to the detrimental effect of face inversion on recognition, face inversion has been shown to have a detrimental effect on the detection of a face embedded within a distracting background [21]. It is thus plausible that the binding of basic visual information (e.g. local orientation information) and the figure-ground segregation of global form is enhanced for upright faces as compared to inverted faces. One way to test this is to measure the visual persistence of global form (henceforth ‘persistence’), a type of short-term perceptual memory that maintains figure-ground segregation in the absence of initial binding cues. Persistence is observed when figure-ground segregation is maintained by the visual system following the removal of an initial binding cue, such as motion. This phenomenon was introduced by Regan [22] and is demonstrated here: http://www.physpharm.fmd.uwo.ca/people/vilis/StopVanishDemo.swf (also see Fig. 1a). The duration of persistence measured via subjective report has been corroborated by neural evidence of persistence measured with fMRI [23], [24], [25], [26]. These studies consistently showed persistence-related activity in object-selective lateral occipital cortex (LO), an area that is known to mediate the binding of early visual information into representations of global form [27], [28]. More specifically, these studies showed that the duration of elevated fMRI activity in LO was consistent with the duration of persistence measured behaviorally. Two of the studies [25], [26] showed persistence-related activity as early as V2, which the authors proposed was due to feedback from LO during figure-ground segregation.

In the current study we investigated the effect of face inversion on persistence. An effect of face inversion on persistence would mean that experience influences figure-ground segregation because holistic processing is the result of extensive prior experience with upright faces [6], [29], [30]. If this effect were observed in the fMRI activity of LO and early visual areas, it would imply that experience influences basic form processing via feedback, potentially originating in the FFA. This would be consistent with a recent report of an experience-related feedback effect on the spatial pattern of fMRI activity in early visual areas [31]. Alternatively, it could be due to shape-based learning, which has been shown to modify the processing of global visual form in LO and early visual areas [32], [33], [34]. In either case, the observation of an inversion effect on persistence would demonstrate an effect of experience-dependent feedback on the visual perception of global form.

In addition to a behavioral measure of persistence, we used fMRI method to measure the effect of face inversion on persistence in LO, two face-selective visual areas—the FFA and the occipital face area (OFA) [35]—and early visual areas (V1, V2, V3 and V4v). An inversion effect on persistence-related fMRI activity in the FFA and OFA would suggest that, in addition to LO, these areas mediate a feedback effect on sustained figure-ground processing in early visual areas; this would also suggest that LO is either modulated by the FFA and the OFA, or is itself a primary basis of the face inversion effect on persistence. An inversion effect on persistence-related fMRI activity in V1 would mean that even the earliest cortical level of visual processing is influenced by feedback from higher-tier visual areas during figure-ground segregation. Finally, we included animals in our study because, like faces, animals are biological forms that have a canonical upright and may also be subject to an inversion effect on persistence. If so, this would suggest that persistence benefits from configural processing, which is disrupted by inversion, and is not limited to faces.

## Materials and Methods

### Subjects

We scanned twelve healthy volunteers (6 female, 6 male; ages 21–40). All participants gave written consent and all experiments were approved by the University of Western Ontario Ethics Review Board.

### fMRI procedure

We performed our experiments using a 3-Tesla Siemens Magnetom Tim Trio imaging system. In both experiments, blood-oxygen-level-dependent (BOLD) data were collected using T2*-weighted interleaved, single segment, echo-planar imaging (EPI), PAT = 2, and a 32-channel head coil (Siemens). In each experiment, the parameters for obtaining functional data were: FOV = 240 mm×240 mm; in-plane pixel size = 3×3 mm; TE = 30 ms; TR = 2 s (single shot), except for our main visual persistence experiment described in the next section (TR = 1 s); volume acquisition time = 2 s; FA = 90°; 36 slices (slice thickness = 3 mm) except for the persistence experiment (18 slices). Functional data were aligned to high-resolution anatomical images obtained using a 3D T1 MPRAGE sequence (TE = 2.98 ms; TR = 2300 ms; TI = 900 ms; flip angle = 15°; 192 contiguous slices of 1.0 mm thickness; FOV = 192×256 mm^2^). Subjects viewed, through a mirror, images that were back-projected onto a screen. The display extended 45° horizontally and 20° vertically. In all experiments the subjects fixated centrally on a stationary dot.

### Visual persistence experiment

Using an event-related design, we performed 4 functional scans per subject with 20 epochs per scan, each epoch lasting 19 s. Subjects viewed stimuli constructed similarly to those used in previous studies of visual persistence form [23], [24], [25], [26], which capitalized on the phenomenon introduced by Regan [22], described earlier. Drawings of faces or animals (∼5° in diameter) were comprised of discontinuous line segments and were superimposed on a background (7°×6°) of randomly oriented line segments (Figure 1a). When these drawings were stationary, subjects confirmed that these were not distinguishable from the background. Following a stationary fixation period of 0.4 s at the beginning of each trial, the drawing (i.e. the figure but not the background of randomly oriented lines) rotated clockwise 15° and counterclockwise 15° in alternation for 3 s. At this point movement stopped and the segments comprising the figure either remained (Persist) or disappeared (Vanish); in both cases the background segments remained for an additional 15.6 s. Subjects indicated with a button press the time at which the figure disappeared; we obtained behavioral data for ten of our twelve fMRI subjects.

We employed two trial-type conditions (Vanish, Persistence), two stimulus categories (faces, animals) and two stimulus orientations (upright, inverted). Our design was not balanced between the Vanish and Persistence conditions in that upright and inverted figures were distinguished for Persistence trials but not Vanish trials (for which there were half the number of trials). We thus denote our conditions as follows: Vanish_faces_; Vanish_animals_; Persist_upright faces_; Persist_inverted faces_; Persist_upright animals_; and Persist_inverted animals_. Subjects participated in four scans each during which all conditions and stimulus types (i.e., 20 different epochs) were randomly permuted and counterbalanced. Stimuli were distributed randomly across the 20 epochs. We used ten face stimuli and ten animal stimuli (the same stimuli were used in upright and inverted conditions); the two categories were highly schematic and trivial to distinguish categorically (See Figure S1). All faces were unfamiliar faces but easily recognizable as faces. Animals were likewise easily recognizable as animals, but not necessarily identifiable at a more specific category level. The Vanish condition occurred four times per scan: two faces (one upright and one inverted) and two animals (one upright and one inverted). A background of randomly oriented lines was present throughout the scan but changed at the beginning of each epoch. All conditions occurred in a pseudo-random order such that no condition or image repeated more than twice in each scan.

### FFA, OFA and LO localizer scans

In addition to measuring persistence using fMRI, we identified category-selective visual areas—the FFA, OFA and LO—in a separate experiment, using different stimuli and conditions. We presented subjects with intact 2D gray-scale photographs of faces, places and common everyday objects, which alternated with scrambled versions of the same images. Three functional scans were performed with 19 epochs per scan, and each epoch was 15 s long. Fifteen images were presented in each epoch at 1-s intervals. Subjects performed a one-back matching task.

### Retinotopic mapping and stimulus-area localizer

We obtained retinotopic maps for the left visual field. As in Strother et al. [36], subjects viewed phase-reversing (temporal frequency = 2 Hz), 100% contrast-defined checkerboard wedge (with a spatial frequency of ∼0.85 cycle/°). The wedge stimulus subtended 45° and extended 15° visual angle into the periphery. This wedge started at the 12-o'clock position (90°upright, UVF, apex at center screen) and rotated anti-clockwise to the 6-o'clock position. The duration of each phase-reversing wedge was 2 s, after which the wedge location revolved anti-clockwise around the center of the screen by 15° (resulting in 33% overlap between each wedge and its successor). At the end of each half-cycle (26 s), the wedge returned to the 12-o'clock position. Individual runs consisted of eight half-cycles, each lasting 24 s. We performed 1 to 3 runs for each individual subject.

In addition to identifying retinotopic visual areas, we performed an additional stimulus-area localizer to identify voxels in these areas that responded most strongly to flickering checkerboard stimuli with dimensions similar to those used in our visual persistence experiment. Observers viewed a flickering (2 Hz) checkerboard pattern similar to that use in our retinotopic mapping experiment except that the areas of this pattern was equal to that of the stimuli used in our persistence experiment. The duration of this flickering stimulus was 16 s and alternated with blank fixation periods (also 16 s); this cycle repeated twenty times during an individual scan. Observers participated in at least one scan.

### Image analysis and ROIs

Image analysis was carried out using the Brainvoyager QX software. 3D statistical maps were calculated for each subject based on a general linear model. When necessary, we also used anatomical landmarks to identify our ROIs. In our group analysis, LO was defined as a set of contiguous voxels which showed significantly stronger activation (*P*<10^−4^) to intact versus scrambled objects. Face-selective areas (the FFA and OFA) were defined as those showing significantly stronger activation to faces than to objects, places and scrambled objects (P<10^−4^). For individual ROI analyses, category-selective ROIs included ∼200 voxels. Retinotopic visual areas were identified on surface maps obtained using cross-correlation analysis, which delineated the borders between V1, V2, V3, and V4v in each subject (Note: although LO is organized retinotopically [36], [37], [38], we did not treat LO as a retinotopic visual area in this study). Within each visual area, we additionally defined an ROI corresponding to the maximal response to our persistence stimulus-area localizer (∼125 voxels per ROI; P<10^−4^); all fMRI data from early visual areas was obtained using both retinotopy and the stimulus-area localizer.

## Results

### Behavioral results

**Figure 2:**
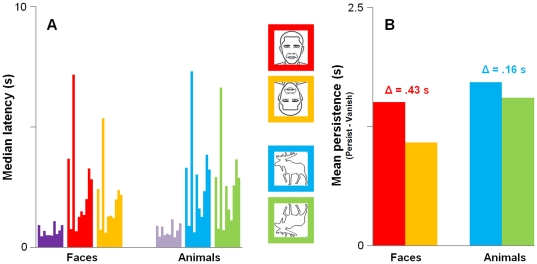
Behavioral results. (A) Median button-press latencies for ten subjects; individual's medians are clustered by condition. Colors correspond to icons for the Persist conditions (red = upright faces; yellow = inverted faces; blue = upright animals; green = inverted animals) and Vanish conditions are purple (no icon; dark purple = Vanish_faces_ and light purple = Vanish_animals_). Latencies were always longer for Persist compared to Vanish. All subjects had longer latencies for upright faces compared to inverted faces; eight of the ten subjects also showed this pattern for animals. (B) Mean behavioral persistence computed as the difference between individual's median latencies for the Persist (animals or faces) and Vanish conditions, each matched by category (red = upright faces; yellow = inverted faces; blue = upright animals; green = inverted animals). The effect of inversion on persistence (Δ) was significant for both faces and animals (p<.05).

The purpose of our behavioral study was to measure the effect of stimulus inversion on persistence. Within-subject paired-samples t-tests (one-tailed) confirmed that latencies for the Persist conditions were always greater than those for the Vanish conditions matched by stimulus category (always p<.05). Median latencies are shown in Figure 2a. Most importantly, median latencies for upright faces were always greater than those for inverted faces in all of our subjects. A corresponding pattern for animals was observed in all but two of our subjects. The group means and standard deviations of individual's median latencies were: Vanish_faces_ = 696.92±220.75 ms and Vanish_animals_ = 718.25±248.59 ms; Persist_upright faces_ = 2204.30±1857.53 ms and Persist_inverted faces_ = 1779.04±1296.88 ms; and Persist_upright animals_ = 2431.38±1907.94 ms and Persist_inverted animals_ = 2270.42±1699.05 ms. Median latencies for the Persist_inverted animals_ condition were more variable across subjects than those for Persist_inverted faces_, which indicates that the effect of inversion on the persistence of animals was less reliable than that for faces. However, this may have been due to uncontrolled stimulus differences between faces and animals.

We next computed a behavioral measure of persistence for each individual by taking the difference between median button-press latencies for the Persistence conditions and those for corresponding Vanish conditions (Persist_upright faces_−Vanish_faces_; Persist_inverted faces_−Vanish_faces_; Persist_upright animals_−Vanish_animals_; Persist_inverted animals_−Vanish_animals_). The group means of this measure are shown in Figure 2b. A repeated-measures ANOVA showed a main effect of stimulus inversion (upright>inverted; F (9, 1) = 5.9, p<.05) and stimulus type (face<animal; F(9,1) = 6.1, p<.05); the difference in the mean durations of persistence (Δ in Figure 2b) was greater for faces (Δ = 425.3 ms) than for animals (Δ = 161.0 ms). The interaction in our ANOVA did not reach significance (F(9, 1) = 4.3, p = 0.07) but again suggested that the inversion effect might be less reliable for animals. In summary, our behavioral results show that inversion interferes with the persistence of both faces and animals.

### Individually-defined ROI analysis: FFA, OFA and LO

**Figure 3:**
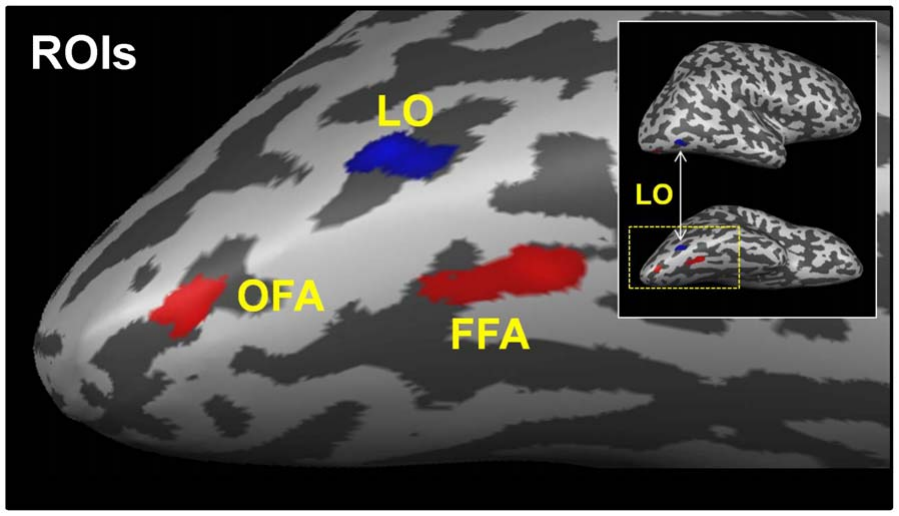
Category-selective ROIs. Three ROIs for a representative subject: object-selective LO (blue) and face-selective OFA and FFA (red).

We analyzed our fMRI data using individually-defined occipito-temporal ROIs (Figure 3), which we identified in all subjects (see Materials and Methods). We chose the FFA and OFA as ROIs because our persistence experiment employed faces; our choice of LO as an ROI was based on previous studies of persistence [23], [24], [25], [26] and its known participation in the domain-general processing of global visual form [27]. Mean Talairach coordinates (x, y, z±SD) for these ROIs were: 37±3, −58±6, −18±4 (FFA), 36±5, −77±4, −16±6 (OFA) and 40±5, −73±4, −9±5 (LO). The coordinates for FFA and OFA were consistent with those reported by Liu et al. [39], and those of LO were also consistent with previously published coordinates [27], [36], [38]. Preliminary analyses showed that a face-selective ROI in the superior temporal sulcus (STS) did not show fMRI evidence of persistence. For this reason, we did not include the STS as an ROI in our analyses.

**Figure 4:**
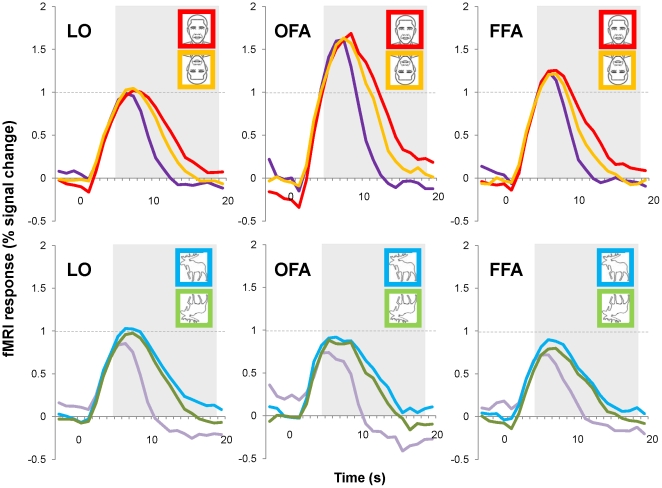
Average fMRI time courses. Event-related averages extracted from individually-defined ROIs. Red and orange time courses for faces are shown in the top row and correspond to upright faces and inverted faces in the Persist condition, respectively (purple = Vanish_faces_). Blue and green time courses for animals are shown on the bottom row and correspond to upright animals and inverted animals in the Persist condition, respectively (purple = Vanish_animals_). fMRI responses were highest to faces in the OFA and the FFA. fMRI activity in all three ROIs remained elevated for Persist conditions relative to Vanish conditions for both faces and animals. Additional elevation of fMRI activity was also apparent for upright stimuli, indicating a facilitation of persistence for upright faces and animals. The grey area indicates the time period over which our fMRI measure of persistence was computed.

We obtained event-related averages from the persistence experiment for each individual's ROIs (Figure 3) and then averaged these across the twelve subjects (Figure 4). All of these time courses show an increase in activation corresponding to the motion of the figure and a decrease shortly after the cessation of motion. The Persist condition time courses show a more gradual decay than those of the Vanish condition, which was expected given our behavioral results and fMRI results from previous studies of persistence [23], [24], [25], [26]. In addition to this persistence effect, an additional effect of stimulus inversion on persistence is also evident in Figure 4, for all ROIs: the decay of time courses is more prolonged for upright as compared to inverted stimulus conditions. This aspect of the event-related averages is strikingly similar to our behavioral results in Figure 2, which showed longer persistence latencies for upright faces and animals than their inverted counterparts.

In addition to the differences in the time courses related to persistence, it is also evident in Figure 4 that fMRI activity in the OFA and the FFA (but not LO) was substantially greater in response to faces than animals (the time courses for faces in the OFA and the FFA cross the horizontal reference line in Figure 4); this was expected since these areas are face-selective. This suggests that there may be two, possibly separable, components of the time courses in Figure 4 that are of particular interest: the initial fMRI response (which appears to reflect face-selectivity) and the magnitude of fMRI activation during the decay of this initial response (which reflects persistence and may also reflect face-selectivity). Before we analyzed these components of our data, we conducted a functional ANOVA [40] to determine whether or not the divergence of the event-related averages for the Persist and Vanish conditions occurred at similar times for faces and animals across the three ROIs. We also ascertained the point at which time courses for faces and animals diverged in the face-selective ROIs. It was important to determine whether or not the temporal aspects of persistence were variable between our ROIs to verify the appropriateness of using the same temporal cut-offs in all of our ROIs in subsequent analyses of these time courses.

The results of our functional ANOVA showed that time courses for the Persist and Vanish conditions diverged maximally by 11 s in all three ROIs (always p<10^−2^), which means that persistence began over six seconds after the offset of motion and does not reflect motion per se [25]. In contrast, face-selectivity was apparent in the divergence of face and animal time course as early as 6 s into the trial (always p<10^−2^), which suggests that face-selectivity was initiated as soon faces began to rotate with respect to the background (assuming a hemodynamic delay of ∼5 s).

In short, the time course of neural persistence suggests an early component that reflects a high degree of face-selectivity and a later component that reflects persistence in the absence of a strong motion cue to the segregation of visual form. We suspected that whereas the early part of our fMRI time courses reflects stimulus selectivity, the latter part reflects the maintenance of global form processing because identification (face or animal) had already occurred. We further tested this hypothesis by conducting separate analyses of the subjects' maximal (henceforth ‘peak’) fMRI responses and the more prolonged fMRI responses acquired during the same trials.

### Peak fMRI response and face-selectivity

**Figure 5:**
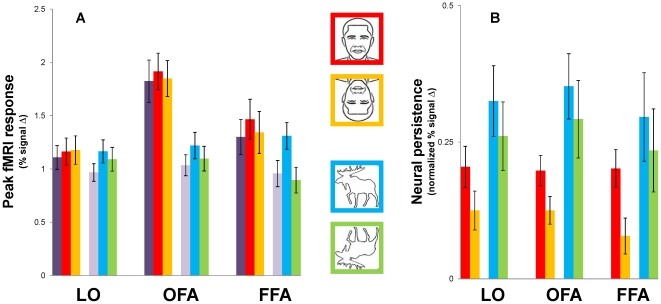
Face-selectivity and persistence-related fMRI activity. (A) Peak fMRI responses to faces were higher in face-selective OFA and FFA as compared to object-selective LO; to a lesser degree, upright faces and animals showed higher peak fMRI responses than their inverted counterparts in the OFA and FFA but not LO. (B) Neural persistence was computed by subtracting fMRI responses to Persist trials from Vanish trials, matched by category, for the period indicated by the shaded area in Figure 4. Neural persistence was higher for upright faces and animals than their inverted counterparts, in all ROIs, even after similar differences in the peak (B) were taken into account. This pattern of results (B) was found in all three ROIs and closely resembles that observed in the behavioral results shown in Figure 2. Error bars show the standard error (SE) for the group (n = 12).

In order to compute a measure of peak fMRI response across individuals and conditions, we identified BOLD maxima taken from individuals' event-related averages. The timing of these maxima varied slightly between subjects (±4 s) but always occurred prior to the point of maximal divergence reported earlier for the Persist and Vanish conditions (Note that these maxima were not the same as those depicted in Figure 4 for the group time courses because the maxima used here did not necessarily occur at exactly the same time for each individual. The averaged values shown in Figure 4 were computed from data that were matched temporally across subjects.) We then averaged these maxima across subjects to obtain the mean peak fMRI responses shown in Figure 5a.

In Figure 5a, the most obvious difference between the peaks for the different conditions can be seen in the face-selective ROIs, which showed greater responses to faces than to animals; this difference appears greater for the OFA as compared to the FFA. We therefore conducted ANOVAs to see whether face-selective cortex responded more strongly to faces than animals, and more strongly to upright faces as compared to inverted faces, which has been reported in other fMRI studies [12], [41], [42], and interpreted as evidence of face-specific visual processing.

We conducted two repeated-measures ANOVAs, each with ROI (LO, OFA, FFA) as a factor, and as additional factors, the Vanish (faces or animals) and Persistence (faces or animals) conditions, each matched by orientation (upright or inverted) within a given ANOVA. Both ANOVAs showed a strong main effect of stimulus category such that peak fMRI responses to faces were greater than those to animals (F(11,1) = 49.1, p<.001 for upright stimuli; F(11,1) = 52.8, p<.001 for inverted stimuli), and also a modest main effect of ROI (F(11,2) = 3.7, p = .042 for upright stimuli; F(11,2) = 3.3, p = .056 for inverted stimuli), suggesting that peak fMRI responses to our stimuli were not the same across ROIs. This was more apparent in the interaction of category with ROI (F(11,2) = 18.2, p<.001 for upright stimuli; F(11,2) = 17.3, p<.001 for inverted stimuli), with face-selective ROIs showing the greatest overall response to face stimuli, as evident in Figure 5a. This confirms that although faces elicited a greater overall response than animals in all of our ROIs, this difference was greatest in the OFA, followed by the FFA, and then by LO. Additionally, we observed a main effect of Persist versus Vanish conditions in both ANOVAs (F(11,1) = 12.9, p<.01 for upright stimuli; F(11,1) = 7.4, p<.05 for inverted stimuli), such that peak fMRI responses for Persist trials were greater than those for Vanish trials. This could be due to the intermixing of upright and inverted stimuli in the Vanish condition (if peak responses to upright stimuli were higher than those to inverted stimuli; see upcoming ANOVA); it could also be due to the offset of the figure elements in the Vanish condition. In either case, it suggests that the peak fMRI response should be normalized when we compute our fMRI measure of persistence.

Because the first two ANOVAs did not test the effect of stimulus orientation directly, we conducted a third ANOVA with the following factors: ROI, stimulus orientation (upright or inverted) and category (faces or animals); unlike the first two ANOVAs, this one did not include data from the Vanish condition. This analysis again showed a significant effect of category (F(11,1) = 48.7, p<.001), where responses to faces were greater than those to animal, and nearly significant main effects of ROI (F(11,2) = 3.1, p = .068) and orientation (F(11,1) = 4.1, p = .067). Both category and orientation showed significant interactions with ROI (F(11,2) = 22.3, p<.001 for ROI×category; F(11,2) = 4.5, p<.05 for ROI×orientation), which supports the expected observation that the face-selective ROIs (FFA and OFA) would show greater face-selectivity than LO in terms of the magnitude of fMRI responses to faces as compared to animals and also to upright faces as compared to inverted faces.

Paired-samples t-tests (two-tailed) showed that responses in the OFA to upright faces were higher than those in FFA (t(11) = 2.33, p<.05), but the two ROIs did not differ in their fMRI responses to upright animals. This suggests that the OFA exhibited greater face-selectivity than the FFA in our persistence experiment. This is important to note because fMRI studies of the effect of face inversion on recognition (e.g., [12]) have reported the opposite. It is possible that because our task did not require explicit recognition, the OFA played a larger role than the FFA. This would support the view that the OFA is more closely related to the visual analysis of face stimuli than the FFA [43], but it would imply that this analysis need not be in the service of face individuation and recognition. Most importantly, the results of all of these analyses suggest that we take into account differences in the peak fMRI responses during our different experimental conditions when we compute our fMRI measure of persistence.

To summarize, we again found evidence of face-selectivity in the peak fMRI responses obtained for face-selective cortex. That is, our peak analysis of the data in Figure 5a corroborated the differences in the peaks shown in Figure 4; face-selective areas showed greater BOLD responses to faces than animals. We also found that face inversion corresponded to decreased fMRI peaks in face-selective cortex but not LO, whereas animal inversion decreased the peak fMRI response in all three of our ROIs (and responses to Vanish were consistently among the lowest, which suggests that peak fMRI responses may also reflect the offset of early visual information present in the line segments corresponding to the figure). However, these differences were quite small compared to the effect of category (face or animal) between ROIs, which is especially evident upon visual inspection of the results for LO and the OFA (Figure 5a).

### Persistence in higher-tier visual areas

As in previous fMRI studies of persistence [25], [26], we normalized individual's event-related averages for each condition to equate for individual differences in overall fMRI response, and also to take into account the observed differences in the peaks related to face-selectivity. We divided values at each time point by the peak percent signal change value for that time course, yielding a maximum value of 1.0 for each condition. We then computed the average of these normalized values for time points occurring 2 s after the offset of our 3-s motion cue (to take in account the hemodynamic lag) until the end of the trial (i.e., from 5 s to 19 s; highlighted in Figure 4). Our measure of neural persistence therefore reflects the decay of the fMRI signal after differences in the magnitude were taken into account. In congruence with our behavioral measure of visual persistence, we then subtracted the average from each of the category-matched vanish conditions (faces/animals) from the corresponding averages for our persistence conditions.

Figure 5b shows the average neural persistence for each condition in our three ROIs. The trend observed in all three of our ROIs is the same as that shown for our behavioral data (Fig. 2), such that animals persisted longer than faces and upright stimuli persisted longer than their inverted counterparts. A repeated-measures ANOVA with ROI, stimulus orientation (upright or inverted) and category (faces or animals) showed main effects of stimulus orientation (F(11,1) = 20.5, p<.001) and category (F(11,1) = 6.7, p<.05) on persistence but no main effect of ROI and no significant interactions. That is, upright stimuli persisted longer than their inverted counterparts and animals persisted longer than faces, in all ROIs. This pattern is commensurate with the results of the ANOVA performed on our behavioral data and suggests that our neural measure of persistence is a valid indicator of the duration of persistence measured behaviorally (Figure 2). We next sought to determine whether this pattern would also be observed in early visual areas.

### Persistence in early visual areas

Although our whole brain analysis did not show significant voxels in early visual areas (Fig. 3; P<10^−2^), previous studies [23], [24], [25], [26] showed that neural persistence occurs in retinotopic cortex. We therefore extracted event-related averages from individually-defined retinotopic areas V1, V2, V3 and V4v, for ten of our twelve subjects (two of our twelve subjects in the whole-brain analysis had incomplete coverage of their early visual areas). In order to restrict our analyses to portions of retinotopic visual cortex corresponding to the retinal extent of our persistence experiment stimuli, we extracted event-related averages from our persistence stimulus-area ROIs described earlier, defined for each early visual area (these ROIs included activation from both dorsal and ventral divisions for V1, V2 and V3).

**Figure 6:**
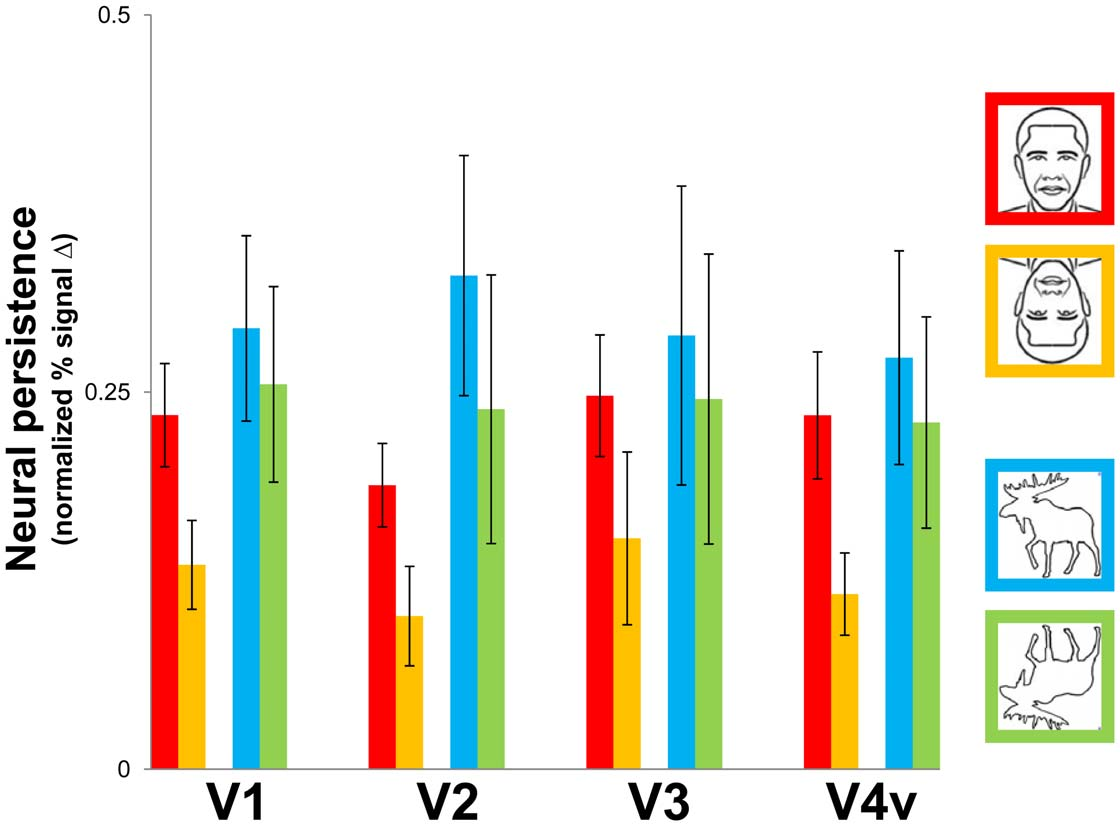
Persistence-related activity in early visual areas. Neural persistence was higher for upright faces and animals than their inverted counterparts, in all early visual ROIs. These fMRI results closely resemble those shown in Figure 5b and the behavioral results in Figure 2. Error bars show the standard error (SE) for the group (n = 10).

None of our early visual ROIs showed significant effects of our conditions on peak fMRI response. We nevertheless computed an fMRI measure of persistence in these areas (Figure 6) using the procedure described earlier for FFA, OFA and LO. We performed the same ANOVA described earlier for the category-selective ROI analysis of persistence. We again observed a significant effect of stimulus orientation (F(9,1) = 18.5, p<.01) but the main effect of category was not significant (F(9,1) = 2.4, p = .16), which suggests that persistence in early visual areas was more sensitive to inversion than object category. As reported earlier for LO, OFA and FFA, we again did not observe a significant main effect of ROI (i.e., effects were similar across the early visual ROIs) and the interaction of stimulus category and orientation was not significant.

To summarize, although we observed some differences in the statistics for data obtained from early visual areas as compared to those observed in our category-selective ROIs (Figure 6), we again found the same effect of stimulus orientation on neural persistence for faces and overall greater persistence for animal stimuli, a pattern of results that was also observed in our behavioral data (Figure 2). The parallels between the results from our early visual ROIs and our category-selective ROIs strongly suggest that higher-tier areas work together with early visual areas to maintain perceptual organization in the absence of strong bottom-up cues.

### fMRI-behavioral measure correlations

In order to assess the within-subject relationship between fMRI activity and our behavioral measure of persistence we performed (Pearson) correlations of individual's fMRI persistence measure and their behavioral results in three stages: (1) we computed correlations for persistence in general; (2) persistence×condition; and (3) the inversion effect on persistence.

We correlated persistence in general by taking each individual's button-press latencies and correlating these with their corresponding fMRI persistence measures obtained for each ROIs, including those in early visual areas (recall that both our behavioral and fMRI measures of persistence were computed within-subject by subtracting Vanish from Persist results). We observed a high degree of correlation in all ROIs (always r>.67 in our category-selective ROIs; and r>.60 in all early visual ROIs) and this correlation was always statistically significant (always p<.01). This finding directly confirms that our behavioral measure of persistence was consistently reflected in our fMRI results.

Next we performed similar correlations broken down by our object category and orientation conditions (i.e., not collapsed across the Persist conditions). We first examined correlations for animals and faces collapsed across orientation (upright or inverted). This approach again revealed high correlations in all of our ROIs (all p<.01 unless otherwise stated). These correlations were highest in our occipito-temporal ROIs and always higher for animals (always r>.76) than faces (always r>.60), which suggests that although our animal persistence results were more variable across subjects; these variable behavioral results were accurately reflected in our fMRI measure of persistence. The most striking aspect of this set of correlations was the observation of high correlations as early as V1 (r = .63 for faces, p<.05; r = .65 for animals, p<.05); high correlations were also observed in the other early visual areas (r ranged from .53 to .70 for faces, and from .62 to .78 for animals, always p<.05). Thus, our behavioral measure of persistence predicts neural activity throughout the ventral visual hierarchy, even as early as V1, in individual subjects.

When we broke down our correlations further by taking into account orientation (upright or inverted), we still observed significant correlations (r ranging from .66 to .92; always p<.05) in all but one ROI for at least three of our four conditions (correlations were also generally high for the remaining condition but did not reach statistical significance). The exception to this was V1, which did not yield any significant correlations for within-subject persistence broken down by all conditions. For our final correlation analysis we computed the behavioral effect of inversion on persistence for face and animals, within subjects, and correlating it with the corresponding subtraction of fMRI responses, within each subject (i.e., Δ in Figure 2, computed at the individual level for our behavioral and fMRI results). Using this approach we observed only one significant positive correlation, in the OFA (r = .76, p<.05; FFA: r = .50, p = .14).

## Discussion

We found that the persistence of global form was greater for outlines of upright faces than inverted faces, and we also observed a detrimental effect of inversion on the persistence of animals. These behavioral observations were consistent with the pattern of persistence-related fMRI activity in all of our ROIs, including a portion of V1 corresponding to the retinal extent of our persistence stimuli. In the FFA and OFA, fMRI responses to faces were higher than those to animals, which confirmed the face-selectivity of these areas as defined using an independent experiment. However, face-selectivity did not predict the face inversion effect on persistence, which was also observed in LO and in early visual areas. All of our early visual ROIs, including V1, showed equal initial fMRI responses to upright and inverted stimuli, but these responses lasted longer for upright forms. We thus propose that higher-tier visual areas, such as the FFA, OFA and LO mediate figure-ground segregation via feedback to retinotopic cortex, and that the participation of face-selective cortex in this process may not be limited to the visual processing of faces.

### Upright faces and animals persist longest

In contrast to previous studies of persistence [23], [24], [25], [26], our use of inverted stimuli allowed us to compare two persistence conditions to each other (Persist_upright_ versus Persist_inverted_; previous studies could only show Persist>Vanish). We found that Persist_upright_ was greater than Persist_inverted_ for both faces and animals. Although the extent to which inversion influences persistence for the two stimulus categories may not be the same—as in the classic study by Yin [1] that showed a greater effect of inversion for faces than non-faces—our study was not designed to test this. Instead, we used animal stimuli to test whether or not an inversion effect on persistence would be limited to face stimuli; it was not.

Our behavioral results replicate and extend those reported previously, and strongly suggest that orientation-dependent representations of global form influence basic visual processing in the service of figure-ground segregation. It is plausible that this effect is limited to familiar stimuli, at least those that are familiar at a categorical level (recall that our task did not require or elicit recognition at the individual level). If so, this would imply that persistence benefits from experience-based holistic processing in addition to the use of local stimulus cues, such as the relative orientations of the line segments comprising our figures. Our fMRI experiments allowed us to identify the prospective neural bases of our behavioral results.

### Peak fMRI response and face-selectivity

Before we examined fMRI activity related to persistence, we analyzed peak fMRI responses to faces versus animals in our category-selective ROIs. We observed greater peak fMRI responses to faces as compared to animals in the FFA and OFA but not LO (Figure 5a). This comparison confirmed that these areas were indeed face-selective in our persistence experiment, even though our task did not require the discrimination of faces and animals, which one could otherwise argue accounts for the effect of inversion on the persistence of both stimulus types (i.e., due to similar processing demands; also see [41]). Given the spatial resolution of our experiments, it is possible that our face-selective ROIs included body-selective voxels. However, the fact that the FFA and OFA exhibited substantially greater responses to faces than animals suggests that that our face-selective ROIs were predominantly face-selective, not body-selective. Thus, our faces and animals stimuli were distinguished in terms of BOLD magnitude, but only in the FFA and the OFA, as expected. This suggests that face-selectivity and the inversion effect on persistence may be independent since the effect of inversion on persistence was not limited to faces or face-selective cortex.

### The effect of inversion on persistence-related fMRI activity

Our fMRI measure of persistence (which controlled for the differences in peak fMRI responses just discussed) showed a greater sensitivity to inversion than did the peak fMRI responses. The purpose of our fMRI measure of persistence was to quantify differences in neural activity corresponding to a period following the offset of the motion phase (i.e., during the Vanish/Persist phase; Figure 1a). Unlike the pattern of results observed in the peak fMRI responses (Figure 5a), the pattern of results observed in our fMRI measure (Figure 5b) persistence consistently resembled that observed in our behavioral results. The observation of an inversion effect on persistence in V1 is especially remarkable because, to our knowledge, it is the first fMRI observation of a high-level feedback effect on figure-ground processing in V1. The fact that an inversion effect on persistence can be observed at all using fMRI is interesting in its own right because the magnitude of the behavioral effect of inversion on persistence was much smaller than the magnitude of persistence itself. It is therefore even more surprising that the inversion effect on our fMRI measure of persistence was as ubiquitous as the persistence effect itself.

The observation of an inversion effect on persistence in our fMRI results is consistent with a recurrent form-processing network that extends from the earliest stages of visual processing to the level of stored object representations. Previous fMRI studies of persistence [23], [25] failed to find significant persistence-related activity in V1, presumably because the experimenters did not localize the retinal extent of their persistence stimuli they used (i.e., they focused on entire early visual cortical areas, only a small portion of which is modulated during persistence). In a recurrent network architecture [44], only the activity of V1 neurons with receptive fields corresponding to the retinal locations of the figure outlines would be influenced, and would thus diminish the ability to detect feedback effects on processing if one examined a given early visual area in its entirety.

### Neural correlates of persistence and the inversion effect

Although we observed significant behavioral effects of inversion on persistence (Figure 2), latencies were variable between subjects and conditions. We therefore sought to directly assess the correspondence between our behavioral results in Figure 2 and our fMRI results in Figure 5b and Figure 6. To date, only one study of the effect of face inversion on fMRI response has reported significant within-subject correlations of behavioral performance. Yovel and Kanwisher [12] showed that face recognition performance correlates with fMRI response magnitude in both the FFA and the OFA. However, when they correlated the effect of face inversion on recognition, they found that only the FFA showed a significant positive correlation. We therefore adopted their approach and correlated individual's persistence latencies with fMRI persistence (using Pearson correlation).

All of the correlations just reported suggest that persistence is ubiquitous in the ventral visual hierarchy and that our behavioral and neural measures of persistence are highly correlated within subjects. And although we saw some evidence that these correlations may be slightly higher for our animal stimuli, we observed high correlations for all of our conditions. Yovel and Kanwisher [12] were faced with a similar scenario in their fMRI study of the face inversion effect on recognition in that they observed high correlations between face recognition performance and fMRI response in multiple face-selective areas. This led them to conduct a final direct correlation of the face inversion effect: they computed the difference in recognition performance for upright and inverted faces and did the same for the corresponding fMRI responses, and then correlated the two derived measures. When they did this, they found only one significant positive correlation for the effect of face inversion on recognition, in the FFA. They assigned much weight to this outcome and concluded that, even though the OFA showed significant correlations with overall face recognition performance, the FFA is the primary neural basis of the face inversion effect. We, however, found the opposite. We prefer to exercise caution in our interpretation of this statistic, but the logic of Yovel and Kanwisher leads to the conclusion that the OFA is the primary neural basis for the face inversion effect on the persistence of form.

### Conclusion

Overall, our results clearly show that the persistence of global visual forms is reduced for inverted stimuli, both perceptually and neurally. Our results are consistent with a disruption of configural processing by inversion. We therefore propose that, along with LO, the FFA and OFA (but not face-selective STS)—and possibly other category-selective visual areas—mediate orientation-dependent figure-ground processing independent of recognition at the sub-category level. The persistence advantage for upright forms suggests that perceptual organization is facilitated for upright familiar objects, and in this sense the visual system is biased to maintain holistic representations of upright familiar forms, not just upright faces.

There were some differences between our results and those of previous fMRI studies of the face inversion effect (e.g., [12]), such as the stronger peak fMRI responses to faces than animals in the OFA relative to the FFA, which may relate to our task and the possibility that the OFA is more closely involved in the initial visual processing of faces than the FFA [43], [45], [46], [47], [48], [49]; this possibility is also consistent with our final fMRI-behavior correlation analysis which only yielded a significant result in the OFA. Although the eventual lack of significant correlations in V1 (when broken down by orientation) suggests a limit to the effect of global orientation-dependent feedback on early visual processing during persistence, it further supports the view that our effects resulted from feedback originating in face-selective visual areas. The sensitivity of the FFA and OFA to global symmetry and configuration [42], [50], [51], [52] would be useful in figure-ground segregation, and may in part explain the results reported here.

A potential limitation of our approach is that, because we relied on ROI analyses, we may have overlooked additional cortical areas or neural populations that could have been sensitive to our experimental conditions. Indeed, it is highly plausible that our ROIs are part of a more extensive cortical network that mediates figure-ground segregation. For instance, body-selective neural populations—which are known to be interspersed with face-selective neural populations [53], [54]—may also mediate effects of category, configuration and object orientation on figure-ground processing. Further studies are necessary to elucidate this and the role of feedback to early visual areas during ongoing figure-ground segregation.

## Supporting Information

Figure S1 **Stimuli.** The figure stimuli which were superimposed on a background of disconnected line segments in the persistence experiment.
(TIF)
