# Supplementary figures and images for: Face Inversion Reduces the Persistence of Global Form and Its Neural Correlates
Source: PLoS One. 2011 Apr 15;6(4):e18705. doi: 10.1371/journal.pone.0018705 (PMC3078111; doi:10.1371/journal.pone.0018705)

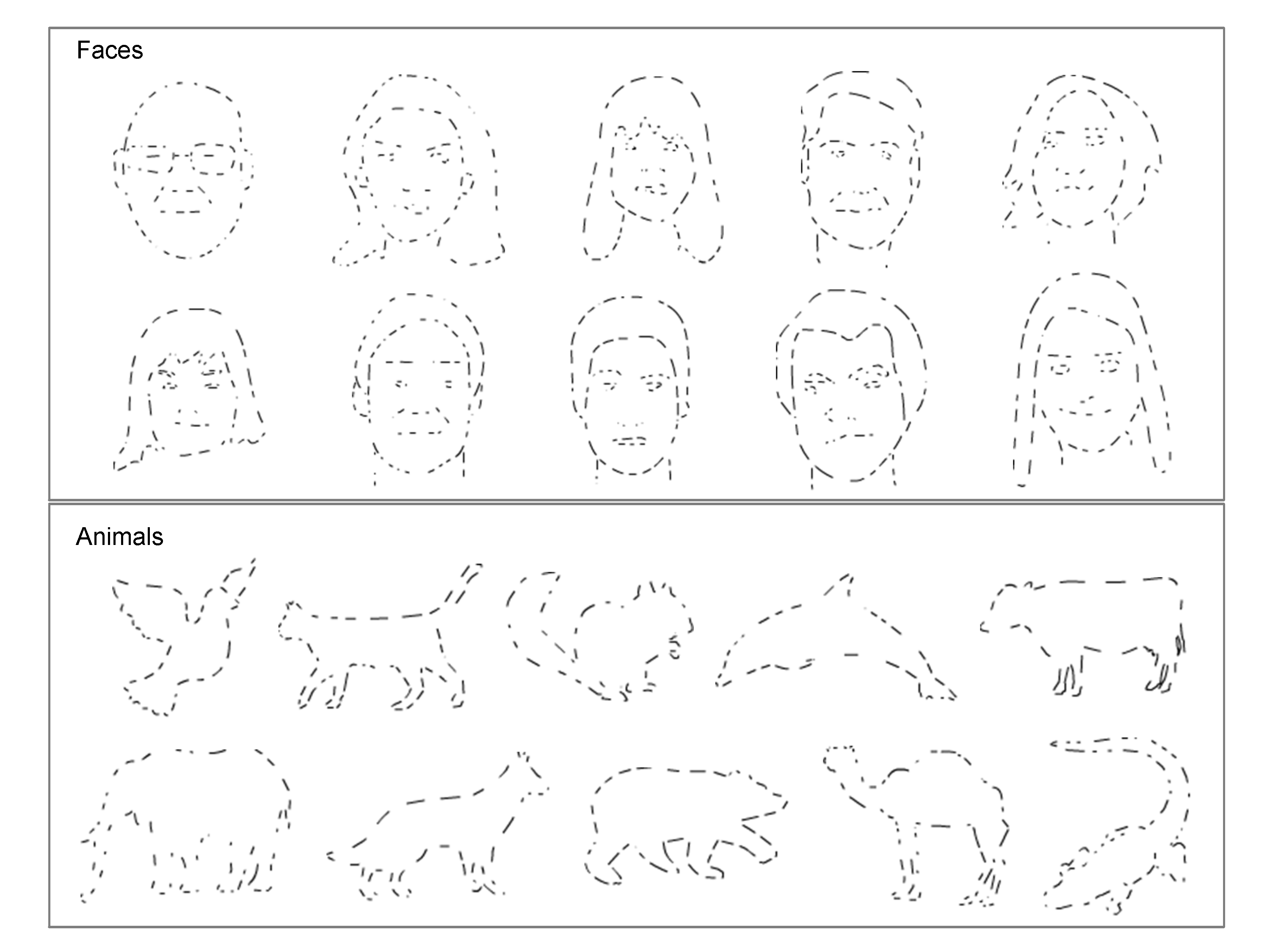

Supplement: Figure S1 — Stimuli. The figure stimuli which were superimposed on a background of disconnected line segments in the persistence experiment. (TIF) [file pone.0018705.s001.tif]
